# Influence of the membrane environment on cholesterol transfer[S]

**DOI:** 10.1194/jlr.M077909

**Published:** 2017-10-18

**Authors:** Jeffrey Michael Breidigan, Natalie Krzyzanowski, Yangmingyue Liu, Lionel Porcar, Ursula Perez-Salas

**Affiliations:** Department of Physics,* University of Illinois at Chicago, Chicago, IL 60607; Large Scale Structures Group,† Institut Laue-Langevin, F-38042 Grenoble CEDEX 9, France

**Keywords:** cholesterol flip-flop, cholesterol exchange, time resolved SANS, lipid vesicles, spontaneous lipid transport, lipid flip-flop, lipid exchange

## Abstract

Cholesterol, an essential component in biological membranes, is highly unevenly distributed within the cell, with most localized in the plasma membrane while only a small fraction is found in the endoplasmic reticulum, where it is synthesized. Cellular membranes differ in lipid composition and protein content, and these differences can exist across their leaflets too. This thermodynamic landscape that cellular membranes impose on cholesterol is expected to modulate its transport. To uncover the role the membrane environment has on cholesterol inter- and intra-membrane movement, we used time-resolved small angle neutron scattering to study the passive movement of cholesterol between and within membranes with varying degrees of saturation content. We found that cholesterol moves systematically slower as the degree of saturation in the membranes increases, from a palmitoyl oleyl phosphotidylcholine membrane, which is unsaturated, to a dipalmitoylphosphatidylcholine (DPPC) membrane, which is fully saturated. Additionally, we found that the energetic barrier to move cholesterol in these phosphatidylcholine membranes is independent of their relative lipid composition and remains constant for both flip-flop and exchange at ∼100 kJ/mol. Further, by replacing DPPC with the saturated lipid palmitoylsphingomyelin, an abundant saturated lipid of the outer leaflet of the plasma membrane, we found the rates decreased by a factor of two. This finding is in stark contrast with recent molecular dynamic simulations that predict a dramatic slow-down of seven orders of magnitude for cholesterol flipping in membranes with a similar phosphocholine and SM lipid composition.

An essential structural component in the membranes of most eukaryotic cells is cholesterol. Cholesterol levels are vital for cellular function, from controlling membrane fluidity and rigidity to directly influencing signal transduction and protein interactions (1). However, cholesterol distribution is very uneven throughout the cell: 60–70% is localized in the plasma membrane (PM), while only 0.01–0.5% is found in the endoplasmic reticulum (2). Although transport of cholesterol between cellular membranes is mostly maintained via vesicular transport and nonvesicular molecular transporters (3), cholesterol’s distribution in the different cellular membranes is thought to heavily depend on the difference in its affinity for various lipid environments. For example, the PM, being rich in saturated lipids is, not surprisingly, where the abundance of cholesterol is highest. Further, cholesterol’s affinity for these membranes will in turn modulate its kinetics in and out of the membranes as well as its movement within the membranes. Indeed, the energetic toll cells have to bear to keep membranes in eukaryotic cells with their unique lipid compositions for specialized functions depends on these affinities and failure to achieve such distributions leads to disease (3–5).

The idea that cholesterol-lipid interactions, which vary according to lipid type, affect cholesterol’s affinity for certain membrane environments has incited numerous studies using model lipid systems. These studies have found that cholesterol interacts preferentially with saturated lipids (6, 7). This finding supports the case of lipid rafts, thought to be nanoscale membrane domains enriched with saturated lipids and cholesterol, and likely implicated as a transduction platform for signaling between membrane proteins (8, 9). However, even if the lipid raft hypothesis has not been demonstrated in cell membranes at physiological temperatures (10), the finding of a greater affinity of cholesterol and saturated lipids explains, in large part, its asymmetrical distribution in the PM. There most cholesterol is found in the saturated lipid-rich outer leaflet of the PM (11) while it is nearly depleted in the cytoplasmic leaflet, being mostly enriched with unsaturated lipids (12). Therefore, even though cholesterol is thought of as highly mobile, flipping quickly between the membrane’s bilayered leaflets (13, 14), this higher affinity for saturated lipids or an environment enriched in saturated lipids could, in fact, slow down cholesterol flipping and, thus, help keep cholesterol asymmetrically distributed across the PM, as recently reported by Liu et al. (11).

Computational studies looking at the behavior of cholesterol in different membrane environments predict a high mobility of cholesterol within the membranes, with flip-flop half-times of tens of nanoseconds to milliseconds (15, 16). However, a dramatic slowdown in the flip-flop rate of cholesterol is predicted when the membrane contains both saturated [palmitoylsphingomyelin (PSM)] and unsaturated lipids (POPC) mimicking a lipid raft; in this case cholesterol takes several minutes (∼30 min) to flip-flop, even though the temperature is well above the miscibility temperature for this lipid mixture (17). Bennett and Tieleman (17), in their all-atom simulations, find that the flip-flop process of cholesterol is hampered by, on the one hand, lesser conformational flexibility imposed by the membrane, and on the other hand, a drop in the number of hydrogen bonds near the membrane center. These two effects contribute to a higher energetic barrier to flip-flop.

Using time-resolved small angle neutron scattering (TR-SANS), we probed the affinity of cholesterol to phos­phatidylcholine (PC) membranes, including mixtures of saturated and unsaturated lipids at temperatures well above the melting temperature (T_m_) of all lipids and the miscibility temperature of the lipid mixtures (18, 19) in order to validate or invalidate the molecular dynamic (MD) predictions. Cholesterol’s affinity with the membrane was investigated in terms of the transfer rates of cholesterol between lipid vesicles (cholesterol exchange) and the transfer rates of cholesterol within the same membrane (cholesterol flip-flop). From these rates, we extracted thermodynamic information for cholesterol transfer, which can also be compared to MD simulation results. In general, we found that the simulations and experiments agree quasi quantitatively in the energy barrier that cholesterol has to overcome to desorb from the bilayer (15–17). However, the energy barrier to cross through the bilayer is found to be much greater in the experiments than in the MD simulations. As a result, MD simulations predict incredibly fast flip-flop rates, in the range of milliseconds or less, while the experiments capture a much slower movement of cholesterol across the bilayer, taking several tens to hundreds of minutes. However, MD simulations have also predicted a seven to eight orders of magnitude slowdown in cholesterol flip-flop in raft-like membrane mixtures containing PSM and POPC (17). When we replaced the saturated lipid dipalmitoylphosphatidylcholine (DPPC) with PSM, we found that there was a slowdown of the cholesterol flipping and exchange by a factor of about two, which is clearly not as dramatic as the simulations suggest (17).

## MATERIALS AND METHODS

### Materials

The 1-palmitoyl(d31)-2-oleoyl-*sn*-glycero-3-phosphocholine (POPC with one of the two tail chains deuterated), 1,2-dipalmitoyl-d62-*sn*-glycero-3-phosphocholine (DPPC with both tail chains deuterated), 1,2-dioleoyl-*sn*-glycero-3-phospho-dioleoylphosphatidylcholine (DOPC), egg sphingomyelin (PSM), and cholesterol were obtained in powder form from Avanti Polar Lipids (Alabaster, AL) and used without further purification. The lipids were used as received. A mini-extruder from Avanti Polar Lipids was used with 1 ml Hamilton syringes for the extrusion of 100 nm unilamellar vesicles (20).

### Preparation of unilamellar lipid vesicles

Initially, lipids and cholesterol were combined with the desired molar ratios. All lipids and cholesterol were used in powder form and dissolved in chloroform once combined. Chloroform was used to ensure the proper mixing of all components. Chloroform was removed by applying a constant stream of nitrogen to the chloroform solution. Vials with dry films were placed in a vacuum oven overnight at 60°C to ensure the complete removal of chloroform. The fully dried mixtures were hydrated with solvents made of appropriate ratios of D_2_O and H_2_O for the particular contrast desired (see the Contrast matching section below). Small unilamellar vesicles were formed by extruding these aqueous solutions through 100 nm polycarbonate filters 41 times at 45°C, which is above the T_m_ of DPPC, resulting in vesicles with nominal 50 nm radii. Two types of vesicles were prepared, one containing only lipids varying in composition containing DPPC or SM and POPC or DOPC (acceptor vesicles) and the other containing cholesterol, at a ratio of 2:1 lipids to cholesterol (donor vesicles) with the lipids being the corresponding lipid combination used in the acceptor vesicles. The stability of the vesicles was verified over time by comparing their small angle neutron scattering (SANS) patterns.

### SANS

SANS measurements were performed on the D22 SANS instrument at the Institut Laue Langevin (ILL) in Grenoble, France, and on the NG7 30 m SANS instrument at the National Institute of Standards and Technology Center for Neutron Research (NIST-CNR) in Gaithersburg, MD. Vesicle characterization was obtained by taking data over a broad Q-range: 0.003 Å^−1^ < Q < 0.6 Å^−1^. For kinetic measurements, a single instrument configuration covering only one order in magnitude in Q (range between 0.005 Å^−1^ < Q < 0.05 Å^−1^) is used. Here Q is the magnitude of the momentum transfer vector, given by Q = 4π sin(θ/2)/λ, where θ is the scattering angle and λ is the neutron wavelength. The wavelength used was 6 Å. To increase the neutron flux, the wavelength spread was set to Δλ/λ = 0.22 and the collimation length was increased to 10 m to reach 0.005 Å^−1^ with a higher beam intensity on the NG7 SANS instrument. Data was collected on a 2D detector and the data was reduced using the reduction packages provided by NIST-CNR and ILL.

### Contrast matching

In these experiments, we were interested in only tracing the movement of cholesterol in different lipid environments; hence, we imposed that the lipid component in the vesicles be invisible to neutrons. This meant that the scattering length density (SLD) of the lipids had to match that of the solvent. The solvents’ SLD was controlled by proper ratios of D_2_O and water (because D_2_O and water have very different SLDs). Each contrast match condition was determined by measuring the intensity patterns from pure lipid vesicles devoid of cholesterol (acceptor vesicles) in various mixtures of D_2_O and water.

For noninteracting vesicles the scattering intensity is given by:

$$I\left(Q\right)=n{\text{v}}_{\text{0}}^{2}{\left(SLD_{vesicle}-SLD_{solvent}\right)}^{2}P\left(Q\right)+\;I_{incoherent}$$

where *P*(*Q*) is, in this case, the form factor for a vesicle, n is the number density of vesicles, v_0_ the volume of one vesicle, and $I_{incoherent}$ is the incoherent background scattering. The contrast match point is obtained by plotting the square root of the background-subtracted scattering intensity as a function of D_2_O volume fraction in the solvent. When the intensity is zero, the SLD of the solvent and the lipids in the vesicles becomes equal (21).

### Cholesterol transfer measurements and modeling

When two populations of vesicles of the same size, one containing cholesterol (donor population) and the other having no cholesterol (acceptor population), are mixed, they will begin to exchange cholesterol and over time the two populations will have vesicles with identical cholesterol compositions. The scattered intensity, which has contributions from the donor and acceptor populations, changes over time as cholesterol moves between the two populations. The captured time evolution of the scattered intensity quantitatively tracks the redistribution of cholesterol between the two populations. Because the shape and size of the vesicles does not change during this process, it is possible to integrate the scattered intensity over all Q from which we obtain the total normalized intensity, given by:

(Eq. 1) $$\tilde{I}\left(t\right)=\frac{I(t)}{I(0)}=\chi_{d}^{2}+\;\left(n_{d}/n_{a}\right)\;{\left(1-\chi_{d}\right)}^{2}$$

where $I\left(t\right)=\int^{\text{​}}\left[I\left(Q,t\right)\;-\;I_{incoherent}\right]dQ$ and where I(Q,t) is the scattered intensity at time *t*. Experimentally, we are able to follow changes in I(Q,t) by using a single instrument configuration covering a Q range between 0.005 Å^−1^ < Q < 0.05 Å^−1^ with data acquisition steps as short as 30 s. A broad wavelength spread, Δλ/λ, of 22% on NG7 SANS at NIST-CNR instrument was used, while a 10% wavelength distribution was used at D22 at ILL.

In equation 1, $n_{d}$ and $n_{a}$ are the respective donor (d) and acceptor (a) vesicle number densities and, in the experiments, were typically equal. The $\chi_{d}$ describes the concentration of cholesterol in the donor population, while $1-\chi_{d}$ corresponds to the concentration of cholesterol in the acceptor population. It is noteworthy to emphasize that the intensity is, in fact, dependent on the square of the concentration of cholesterol in donor and acceptor vesicles. Vesicles, having inner and outer facing leaflets, also have corresponding inner and outer leaflet cholesterol concentrations such that $\chi_{d}=C_{\text{i}\_\text{d}}+C_{\text{o}\_\text{d}}$, where $C_{\text{i}\_\text{d}}$ and $C_{\text{o}\_\text{d}}$, respectively, correspond to the concentration of cholesterol in the inner (i) leaflet and outer (o) leaflet in the donor population. Indeed, these leaflet concentrations in donor (d) and acceptor (a) vesicles are the only parameters that change as a function of time. These time-dependent concentrations of cholesterol in each leaflet are coupled through first order differential equations given by (21):

(Eq. 2a) $$\frac{dC{\left(t\right)}_{i\_d}}{dt}=-k_{f}\left[C{\left(t\right)}_{i\_d}-C{\left(t\right)}_{o\_d}\right]$$

(Eq. 2b) $$\begin{array}{c} \frac{dC{\left(t\right)}_{o\_d}}{dt}=k_{f}\left[C{\left(t\right)}_{i\_d}-C{\left(t\right)}_{o\_d}\right]\\ -k_{ex}C{\left(t\right)}_{o\_d}+k_{ex}^{\prime }C{\left(t\right)}_{o\_a} \end{array}$$

(Eq. 2c) $$\begin{array}{c} \frac{dC{\left(t\right)}_{o\_a}}{dt}=k_{f}\left[C{\left(t\right)}_{i\_a}-C{\left(t\right)}_{o\_a}\right]\\ -k_{ex}^{\prime }C{\left(t\right)}_{o\_a}+k_{ex}C{\left(t\right)}_{o\_d} \end{array}$$

(Eq. 2d) $$\frac{dC{\left(t\right)}_{i\_a}}{dt}=-k_{f}\left[C{\left(t\right)}_{i\_a}-C{\left(t\right)}_{o\_a}\right]$$

where $k_{f}$, $k_{ex}$, and $k_{ex}^{\prime }$ correspond to rates of cholesterol flip-flop and exchange. Indeed, the exchange rates, $k_{ex}$ and $k_{ex}^{\prime }$, are related by: $k_{ex}^{\prime }=\frac{n_{d}}{n_{a}}k_{ex}$ and are equal when $n_{d}=n_{a}$.

However, when flipping rates are not limiting, then equations 2a–2d reduce to:

(Eq. 3a) $$\frac{dC{\left(t\right)}_{\text{d}}}{dt}=-\frac{k_{ex}}{2}C{\left(t\right)}_{\text{d}}+\frac{k_{ex}^{\prime }}{2}C{\left(t\right)}_{\text{a}}$$

(Eq. 3b) $$\frac{dC{\left(t\right)}_{\text{a}}}{dt}=-\frac{k_{ex}^{\prime }}{2}C{\left(t\right)}_{\text{a}}+\frac{k_{ex}}{2}C{\left(t\right)}_{\text{d}}$$

which represent solely an exchange process.

## RESULTS

The passive movement of cholesterol between and within membranes is recognized as key to our understanding of the energetic cost of maintaining lipid composition gradients within the membranes of cells. However, due to variations in the measurement protocols and methodologies, the rates and energetics for lipid movement continue to be contentious after four decades of studies. We have shown that when monitoring chemically altered cho­lesterol, such as ergosta-5,7,9(11),22-tetraen-3β-ol (DHE), which is considered an analog of cholesterol, or in the presence of an extraneous compound, such as cyclodextrin, the results are very different compared with unaltered cholesterol (21). This, we have argued, has in large part contributed to the reported differences in the rates. Hence strategies that do not compromise the chemical identity of cholesterol or the surrounding lipid environment have a clear advantage. Further, in situ measurements, which do not require the separate sampling of the system, can have the additional advantage of capturing cholesterol composition changes in donor and acceptor populations in all stages of the transfer process (22, 23).

**Fig. 1.**
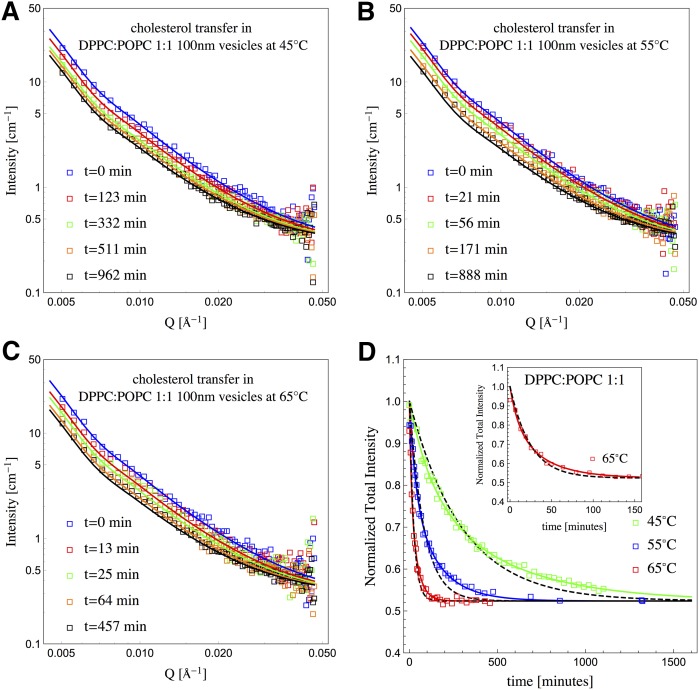
Scattered intensities as a function of time, tracking the movement of cholesterol between donor and acceptor vesicles with lipid membranes composed of DPPC and POPC in a 1:1 ratio at 45°C (A), 55°C (B), and 65°C (C). D: Resulting normalized total intensities as a function of time for these three temperatures and corresponding fits using equation 1. Flipping and exchange rates ($k_{f}$ and $k_{ex}$, respectively) were extracted from the fits using equations 2a–2d. The dashed black curves correspond to fits in which flipping rates are not rate-limiting and changes in intensity are only due to an exchange process (equations 3a and 3b). Inset: enlarged view of 65°C data and fits shown in D. In A–C, the data at *t* = 0 was fitted using equation 5 and a vesicle form factor and shown as a continuous line. For *t* > 0, calculated scattering curves were obtained using equation 4.

TR-SANS can straightforwardly take advantage of these strategies for the study of the passive movement of cholesterol in free-standing membranes (vesicles). In the present study, we investigated the role of the membrane’s environment on cholesterol movement, especially the role of saturation level when mixing saturated and unsaturated lipids. **Figure 1A–C** shows the scattered intensity changes as a result of cholesterol redistributing between donor and acceptor populations in 100 nm in diameter vesicles with membrane compositions consisting of a saturated lipid, DPPC (palmitoyl tails), and an unsaturated lipid, POPC (containing one palmitoyl tail and one oleoyl tail), in a 1:1 ratio at three different temperatures. The temperatures probed were well above the T_m_ of DPPC. The intensities decrease as a result of the movement of cholesterol only, because all lipid contribution to the intensity has been removed via contrast matching of the SLD of the PC membrane to the solvent (SLD_lipids_ = SLD_solvent_). The calculated curves through the scattered intensity data shown in Fig. 1A–C were obtained from:

(Eq. 4) $$I\left(Q,t\right)=\beta \left(Q\right)\tilde{I}\left(t\right)\;$$

where β(Q) is the time-independent prefactor of the normalized total intensity and given by:

(Eq. 5) $$\beta \left(Q\right)=\frac{n_{d}{\text{V}}_{0}}{V}\Delta {\text{SLD}}^{2}v_{0}^{2}P\left(Q\right)$$

where $\frac{n_{d}{\text{V}}_{0}}{V}$ is the volume fraction of the donor population. ΔSLD corresponds to the contrast between cholesterol and the solvent ($\Delta \text{SLD}={\text{SLD}}_{cholesterol}-{\text{SLD}}_{solvent}$) and $v_{0}$ corresponds to the initial volume fraction of cholesterol in the donor vesicles. P(Q) is the vesicle form factor. β(Q) is obtained by fitting the *t* = 0 data. The rates $k_{f}$ and $k_{ex}$ were extracted from fits to the normalized total intensity data using equations 2a–2d in conjunction with $\tilde{I}\left(t\right)$, given by equation 1 and shown in Fig. 1D. Also in the figure are fits (dashed black lines) to the normalized total intensity data using, instead, equations 3a and 3b in which only an exchange process is permitted. Although visually it is clear that a flipping and exchange process provides a better description of the time evolution of the total intensity, it has been argued (24) whether the two-process model is adequate to model the data. In order to validate our analysis, we then performed an Akaike information criterion (AIC) test (25). We wish to select, from among candidate models, the model that minimizes the information loss. How much information is lost by the model in equations 2a–2d (exchange and flipping model) compared with equations 3a and 3b (exchange only model) can be obtained by comparing their AIC values. In all cases, we found lower AIC values for the exchange and flipping model (supplemental Table S1) indicating that there is less information lost in the two-process (flipping and exchange) model compared with the one-process (only exchange) model. Further, we calculated the likelihood that the one-process model was comparable to the two-process model in terms of information content and found it to be extremely low; for the 65°C data presented in Fig. 1D, the likelihood was 0.05 or, equivalently, the two-process model is 20 times better in describing the data than the one-process model, which allows us to discard the one-process model. In supplemental Figs. S1 and S2, we show similar plots corresponding to membranes composed of pure DPPC, as well as a mixture of DPPC and DOPC (1:1). For these data, the AIC test gave values that were lowest for the flipping and exchange model; but also from the comparison between the two models, we could discard the only exchange model for having low likelihoods for comparable information content, all of which were lower than 0.05. Only for DPPC and DOPC (1:1) membranes at 65°C was the likelihood 0.3, which meant that the two-process model was just three times better than the one-process model, and therefore remains a reasonable model to consider. However, overwhelmingly, the flipping and exchange model best described the data. The corresponding exchange and flip-flop rates obtained in different lipid environments at 55°C are tabulated in **Table 1**. Data for the movement of cholesterol in a pure POPC bilayer was previously published (21).

**TABLE 1.** Intra- and inter-membrane diffusion rates and half-lives for cholesterol transport in vesicles composed of phospho choline lipids as well as PC lipids and sphingomyelin at 55°C

For T = 55°C	*k*_ex_ (min^−1^)	*t*_1/2, ex_ (min)	*K*_f_ (min^−1^)	*t*_1/2, f_ (min)
DPPC	0.0028 ± 0.0001 (±4%)	248 ± 10	0.0016 ± 0.0004 (±25%)	433 ± 108
DPPC:POPC	0.0081 ± 0.0005 (±6%)	86 ± 5	0.0042 ± 0.0005 (±13%)	165 ± 21
DPPC:DOPC	0.023 ± 0.002 (±9%)	30 ± 3	0.014 ± 0.004 (±29%)	50 ± 15
DPPC:2 POPC	0.013 ± 0.001 (±8%)	53 ± 4	0.007 ± 0.001 (±14%)	99 ± 14
SM: 2 POPC	0.0056 ± 0.0003 (±5%)	124 ± 6	0.00368 ± 0.001 (±27%)	188 ± 51

Saturated and unsaturated phospho choline membranes (DPPC, DPPC:POPC, DPPC:2 POPC and DPPC:DOPC) and unsaturated phospho choline lipids and sphingomyelin membrane (SM:2 POPC). The error is also presented as a percentage of the estimated value.

**Fig. 2.**
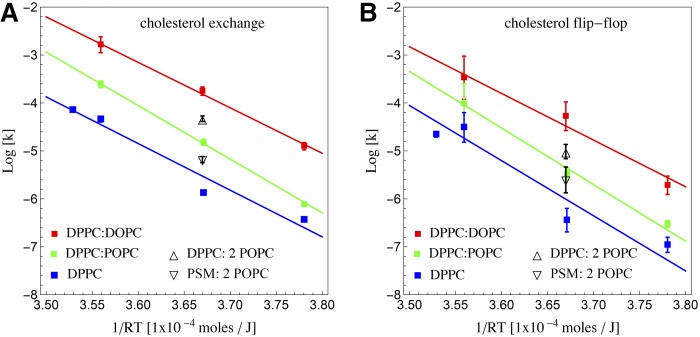
Arrhenius plots for exchange (A) and flipping (B) rates of cholesterol in vesicles composed of saturated lipids with varying percentages of oleoyl tails. In DPPC vesicles, all tails are saturated, while DPPC:POPC vesicles, at 1:1 ratio, contain 25% oleyl tails. Similarly DPPC:DOPC vesicles, at a 1:1 ratio, contain 50% oleyl tails. Rates at 55°C for cholesterol in vesicles of DPPC:POPC and PSM:POPC at a ratio of 1:2 (containing 33% oleoyl tails) are also shown. The lines through the data represent least square fits. The slope represents the energy of activation, $E_{a}$, in each case.

Arrhenius plots of the exchange and flip-flop rates of cholesterol in these lipid environments as a function of temperature are shown in **Fig. 2**. We found that the slowest rates are for the system with no oleoyl tails: in the saturated DPPC membranes. Cholesterol’s transfer rates in DPPC:POPC (1:1) membranes lie between those of DPPC membranes and DPPC and DOPC membranes. In comparison to our work on cholesterol movement in POPC membranes (21), we found that cholesterol in DPPC:DOPC at a 1:1 ratio behaves similarly to when the membrane is POPC. This suggests that, at a given saturation density, cholesterol’s behavior is mostly insensitive to whether there are two POPC neighboring molecules or a DPPC molecule and a DOPC molecule. Hence only the average density of palmitoyl tails to oleyl tails determines the rates of exchange and flip-flop for cholesterol and not the local tail organization. Indeed, and not unexpectedly, it is the addition of unsaturated (oleoyl) tails to the membrane that adds disorder to the membrane as simulations have reported (26). Increased disorder, in turn, induces faster mobility of cholesterol, both for exchange as well as flipping.

From the Arrhenius fits shown in Fig. 2, we also found the activation energy, $E_{a}$, for flipping and for exchange. Because the slopes of the linear fits, representing the activation energy, are very similar because their slopes are nearly parallel. Further $E_{a}$ for exchange and for flipping is very similar (between 100 and 110 kJ/mol). Thermodynamic parameters can be extracted from $E_{a}$ according to Eyring’s transition state theory (27, 28), as implemented by Homan and Pownall (29) in which entropy and enthalpy are related as follows:

(Eq. 6) $$e^{\Delta S^{‡}/R}=\frac{N_{A}h}{RT}\kappa_{T^{*}}e^{-\Delta H^{‡}/RT}$$

where $N_{A},h,R$ are Avogadro’s number, Plank’s constant, and the gas constant, respectively. T is temperature in Kelvins and $\kappa_{T^{*}}$ corresponds to the rate extrapolated to 37°C (310 K). The enthalpy is related to the activation energy by $\;\Delta H^{‡}=E_{a}-RT$ and the difference between the enthalpy, $\;\Delta H^{‡}$, and the entropy term, $T\Delta S^{‡}$, is the free energy: $\Delta G^{‡}=\Delta H^{‡}-T\Delta S^{‡}$. The corresponding thermodynamic parameter values obtained from the activation energies are reported in **Table 2**.

**TABLE 2.** Free energy $\Delta G^{‡}$, the entropic term $T\Delta S^{‡}$, and enthalpy ($\Delta H^{‡}$) for flipping and exchange of cholesterol in PC membranes

For T = 55°C	$\Delta G^{‡}$ (kJ/mol)		$\left|T\Delta S^{‡}\right|$ (kJ/mol)		$\Delta H^{‡}$ (kJ/mol)	
	Flipping	Exchange	Flipping	Exchange	Flipping	Exchange
DPPC	104 ± 36	97 ± 21	9 ± 26	7 ± 15	112 ± 26	95 ± 15
DPPC:POPC	102 ± 14	98 ± 4	13 ± 10	9 ± 3	115 ± 10	109 ± 3
DPPC:DOPC	98 ± 24	96 ± 6	4 ± 17	4 ± 4	95 ± 17	92 ± 4

$\Delta G^{‡}$ varies minimally in the temperature range studied (45–70°C). $T\Delta S^{‡}$ is significantly smaller than $\Delta H^{‡}$ and $\Delta G^{‡}$, suggesting a small entropic contribution. The error corresponds to the error in activation energy $E_{a}$ obtained from the Arrhenius fit, and which is propagated to the other thermodynamic quantities.

## DISCUSSION

The membranes of cells contain a great variety of lipid molecules. Such variety suggests their complex and unique role in cell function. A first step in directed functionality is the establishment of lipid composition differences in different membranes within the cell (2). Lipid functionality is further specialized by the establishment of an asymmetric distribution of lipids across the membranes, with its most notable example being the PM of mammalian cells (2). The distribution of lipids, from the endoplasmic reticulum and Golgi apparatus where they are synthesized (30) to their target membranes, is maintained by mechanisms involving vesicle transport pathways as well as the active and passive transport by lipid transporters (2, 31–33). This distribution of lipids across membranes is crucial for cell homeostasis (2). One of the questions of interest is the energy cost for this distribution. This question can be addressed, in part, by determining the thermodynamic energy barriers and kinetic characteristics of the passive movement of lipids between and within membranes. These define the energetic baseline the cell has to overcome to reach a homeostatic state that is then maintained via ATP and non-ATP processes (14).

Cholesterol is one example for which there is considerable debate regarding how fast it moves between and within membranes, as well as its distribution, particularly across membrane leaflets in the PM. Because it is known that the outer PM leaflet is rich in saturated lipids, it was expected that this leaflet would also be enriched with cholesterol because it shows greater affinity for saturated lipids than it does for unsaturated lipids (1). However, measuring the distribution of cholesterol across the PM has not been straightforward. As recently reviewed by Murate and Kobayashi (34), some reports suggested that cholesterol was, instead, enriched in the inner leaflet of the PM, while other reports suggested that cholesterol was uniformly distributed across the PM because it flip-flops fast. It was not until the recent work by Liu et al. (11) that it was confirmed that cholesterol is asymmetrically distributed across the PM and mostly localized in the outer PM leaflet. Still, in the view of Liu et al. (11), as well as others, transporter proteins control the distribution of cholesterol across the PM because cholesterol’s transbilayer motion is considered to be fast (less than a second) (13, 14, 35, 36).

MD simulations offer atomistic detail of cholesterol’s flip-flop process in membranes. In single lipid PC membrane environments, the simulations show that the trans-bilayer motion or flip-flop of cholesterol is fast, on the order of nano- to milliseconds (15, 16), in apparent agreement with experiments (13, 14, 35, 36). However, recent simulations by Bennett and Tieleman (17), using a mixed membrane environment composed of saturated and unsaturated lipids as a simple model for the outer leaflet of the PM, showed that cholesterol flip-flop slowed down considerably to tens of minutes, suggesting that the lipid environment could also provide an additional mechanism to maintain leaflet asymmetry as required in the PM.

Using TR-SANS, we studied the movement of cholesterol in PC membranes. Previously, we reported that cholesterol moves slowly between POPC membranes (exchange) as well as, and surprisingly, between membrane leaflets (flip-flop), both taking several tens to hundreds of minutes for temperatures ranging between 40°C and 60°C (21). Slow cholesterol flip-flop in POPC, though surprising, was supported by the finding that cholesterol analogues, the presence of extraneous compounds like cyclodextrin, or even the presence of a supporting surface, have a huge impact on lipid transbilayer motion (21, 37, 38). Thus, to study lipid movement in free-standing membranes, only those techniques capable of following tag-free lipid molecules, nonperturbatively and in situ, like TR-SANS, can accurately measure the rates for exchange and flip-flop in membranes.

In the current study, we set about to modifying the membrane environment by varying the ratio of saturated tails to unsaturated tails, yet not varying the headgroup (PC) nor the membrane thickness (39). We found that the rates of exchange and flip-flop of cholesterol in the membranes became gradually slower as the ratio of saturated (palmitoyl) to unsaturated (oleoyl) tails increased. The rates of cholesterol in DPPC were found to be approximately four times slower than in POPC. The behavior of cholesterol in membranes composed of a 1:1 mixture of DPPC and DOPC, which preserved POPC’s ratio of oleoyl to palmitoyl tails, was found to be similar to that found in POPC membranes. Membranes composed of DPPC and POPC mixtures (1:1 and 1:2, respectively) showed rates that were between the fully saturated membrane (DPPC) and the unsaturated POPC membrane (or equivalently, DPPC:DOPC 1:1). In like manner, in DPPC:POPC 1:2 membranes, cholesterol rates were faster than in DPPC:POPC 1:1 membranes. That the flip-flop and exchange rates increase as the number density of unsaturated lipid tails increases in the membrane is not surprising because unsaturation in the tail region adds disorder to the membrane (26). It is interesting that the rate of flip-flop was consistently slower than the exchange rate and nearly constant at ~0.5, independent of the membrane environment. As a result, the energetics for both exchange and flip-flop are unchanged as the membranes transformed from a purely saturated membrane (DPPC) to an unsaturated membrane (POPC or one mimicking POPC).

As shown in Table 2, we were able to obtain the free energy barrier from E_a_ via Eyring’s transition state theory (27, 28), as adapted by Homan and Pownall (29). Indeed, the free energy barriers to exchange and to flip-flop are very similar and similar to the ones found for POPC, which are of order ~100kJ/mol (21). This is reasonable from the perspective that the membranes’ thickness (39) and headgroup region (PC) remained unchanged. In lipids, the membrane thickness and not the saturation level has the most significant effect on lipid desorption and flip-flop energetics, as reported by Sapay, Bennett, and Tieleman (40). The entropic term found is small with its sign unclear given the size of the error bars (negative being unfavorable and positive being favorable). We know that there is certainly a favorable entropy to mix of order ∼2 kJ/mol (∼RTln2), which is consistent with these small values. Also, an entropic conformational contribution from this rigid molecule may be expected to be small. We find that enthalpy is therefore the main contribution to the free energy. In contrast, MD simulations by Bennett et al. (16) found that in DPPC, the entropic term has a more significant contribution to the free energy. In the case of cholesterol desorption, the entropic term is unfavorable and of the order of ∼80kJ/mol while for flip-flop it is favorable and of the order of ∼50kJ/mol. On the other hand, the enthalpy for cholesterol desorption is favorable and of the order of ∼150kJ/mol while it is unfavorable for flip-flop and of the order of ∼70 kJ/mol. This in turn results in a large free energy barrier to desorb (∼75kJ/mol), which is consistent with our experimental results, but a low energy barrier to flip-flop (∼25kJ/mol), which is not consistent with our experimental findings.

As mentioned earlier, Bennett and Tieleman (17), in their MD simulations study, showed a striking change in the flip-flop rates of cholesterol and the energy to flip when using a mixture of a saturated lipid and an unsaturated lipid in their membranes consisting of PSM and POPC in a 1:1 ratio. Bennett and Tieleman (17) obtain a slowdown of several orders of magnitude compared to single lipid systems.

As seen in Fig. 2 (and supplemental Fig. S3), our results show that the flip-flop and the exchange rates of cholesterol slow down in a PSM-containing membrane compared with a similar, but DPPC-containing membrane [the ratio being 1:2 saturated to unsaturated lipids, at 55°C, which is well above the miscibility temperature for these mixtures (19)]. However, the slow down is only by a factor of order 2. Slower exchange and flip-flop rates in the presence of PSM agrees with the report by Sankaram and Thompson (41) that suggests that PSM interacts more strongly through its amide group (dipolar in character) with cholesterol’s amine polar group compared with DPPC’s ester linkage. Or as more recently investigated by Yasuda et al. (42) and Wang and Klauda (43) or previously reported by Brown (44), cho­lesterol also seems to be able to break PSM’s hydrogen bond network and interdigitate between PSM molecules more efficiently, while less so in DPPC. Hence, our experimental results can be explained by a molecular PSM-cholesterol interaction rather than a synergistic effect due to the mixture of PSM, POPC, and cholesterol, as suggested by Bennett and Tieleman (15).

In closing, there is no doubt that MD simulations are an essential tool to understanding lipid behavior in membranes, clearly complementing experimental work with atomistic structural detail as well as energies that can be verified by experiments (45). Indeed, this work not only shows that there is some agreement between experiments and simulations but also shows that there are still significant differences and, therefore, there is need for this essential feedback loop between the two.

## CONCLUSIONS

Using TR-SANS, a noninvasive in situ technique that is able to track the movement of unaltered lipids and sterols, we obtained the flip-flop and exchange rates and energetics of cholesterol in PC membranes consisting of mixtures of saturated palmitoyl (16:0) and unsaturated oleolyl (18:1) tails. Starting from a fully saturated membrane (DPPC), we found that as the number of unsaturated (oleoyl) lipid tails increases in the membrane, both flip-flop and exchange rates of cholesterol increase. The maximum ratio of oleoyl tails to palmitoyl tails studied was 1:1 in a mixture of DPPC and DOPC. For cholesterol, this membrane environment was found to be equivalent to POPC, for which we had reported earlier (21). The half-time for cholesterol flip-flop and exchange in all cases was found to be slow, taking several tens to hundreds of minutes in the temperature range between 45°C and 70°C, which is well above the melting transition temperature of DPPC. The rate of flip-flop was consistently slower than the exchange rate and nearly constant at ∼0.5, independent of the membrane environment. From the thermodynamic analysis of the rates, we found that the free energy barrier to move cholesterol across these PC membranes of similar thickness remains constant for both flip-flop and exchange, both at about 100 kJ/mol. Because enthalpy and the free energy were found to be comparable, the entropic term is found to be small, which is not surprising given that cholesterol has a more rigid structure than PC lipids with long hydrocarbon tails.

Interestingly, we found that by exchanging the fully saturated lipid DPPC with PSM, which have similar T_m_ values (18), we detected a slowdown in both flip-flop and exchange by a factor of order two. This comparison was made at 55°C, well above the miscibility transition temperature of either mixture (19). This is explained by a well-established stronger cholesterol/PSM interaction as compared with cholesterol/DPPC (41–44). As a result, this makes cholesterol less likely to flip or desorb from PSM-containing membranes than from DPPC-containing membranes.

Results from MD simulations studying cholesterol’s movement in similar membrane environments, as reported here, find that cholesterol flip-flops fast, with half-times from the nano- to millisecond time scale (15, 16), except when the lipid environment consists of PSM and POPC, where the flip-flop process dramatically slows down by several orders of magnitude, from microseconds to tens of minutes, even when the membrane is well above the miscibility temperature for the mixture (17). This was clearly not observed in our experiments.

In terms of the energetic barriers to desorb and flip, MD simulations find that the energetic barrier for cholesterol to desorb from the membrane is very similar to what we have obtained experimentally here and previously (∼90 kJ/mol for the simulations (15) and ∼100 kJ/mol for the experiments) (21). However, the energetic barrier to flip-flop is significantly shallower in the simulations than what we find in the experiments (∼20 kJ/mol for the simulations and ∼100 kJ/mol for the experiments) resulting in fast flip-flop in the simulations and slow flipping in the experiments (15, 21).

The study of the passive movement of lipids, as well as cholesterol, in different lipid environments is of interest to determine the energetic baseline that the cell has to overcome to achieve lipid homeostasis (33). Though it is widely agreed that lipids flip slowly (several hours), cholesterol as well as other sterols are thought to move fast through a bilayer (<1 s) (13, 14, 35, 36). However, our TR-SANS measurements suggest that, in free-standing membranes, the transbilayer movement or flip-flop of cholesterol is much slower, taking several tens to hundreds of minutes. We have explored possible contributors to discrepancies in the reported rates from different experimental techniques and have found that tagging or altering the environment of the membrane with extraneous molecules, like cyclodextrin, suggest a behavior for cholesterol that is not found in untagged and unaltered environments (21). More recently, we found how defects in membrane packing induced by a supporting surface facilitate lipid flip-flop too and methodologies that can potentially have this effect on membranes result in inaccurate measured rates (39). From the MD side, we have also suggested that the tendency of the simulations to accumulate cholesterol in the bilayer center suggests that the energy barrier to flip is too low (46, 47). Further, with TR-SANS, we have demonstrated that although exchange and flip-flop are coupled, it is their interdependence in the movement of lipids between and within membranes that allows for their distinction, even when the most significant contribution in TR-SANS comes from the exchange process as supported by the AIC test. Of course, when the flip-flop rates are faster than those of exchange, such a distinction cannot be made using TR-SANS, as we recently discussed (37).

Finally, we did not find an enhanced decrease in the flip-flop rate in membranes consisting of mixtures of saturated and unsaturated lipids compared with the pure lipid systems, but instead found a systematic decrease in the rates with saturation content in the membranes. The inclusion of PSM does, however, have a stronger slowing effect than DPPC. Together these results suggest that lipid composition in membranes can play an important role and energetically assist in an asymmetric distribution of cholesterol in the PM, where most of the cholesterol is localized in the PSM-rich exocellular facing leaflet (11).

## Supplementary Material

Supplemental Data
