# Supplementary material for: Influence of the membrane environment on cholesterol transfer
Source: J Lipid Res. 2017 Oct 18;58(12):2255–63. doi: 10.1194/jlr.M077909 (PMC5711489; doi:10.1194/jlr.M077909)
Supplement: Supplemental Data [file supp_58_12_2255__index.html]

Influence of the membrane environment on cholesterol transfer — Influence of the membrane environment on cholesterol transfer — Supplemental Data 

# Influence of the membrane environment on cholesterol transfer

## Supplemental Data

- Supplemental Information (.pdf, 2.1 MB) - Supplemental Information, with figures and tables
