# Supplementary material for: Influence of the membrane environment on cholesterol transfer
Source: J Lipid Res. 2017 Oct 18;58(12):2255–63. doi: 10.1194/jlr.M077909 (PMC5711489; doi:10.1194/jlr.M077909)
Supplement: Supplemental Data [file 10.1194_M077909_jlr.M077909-1.pdf]

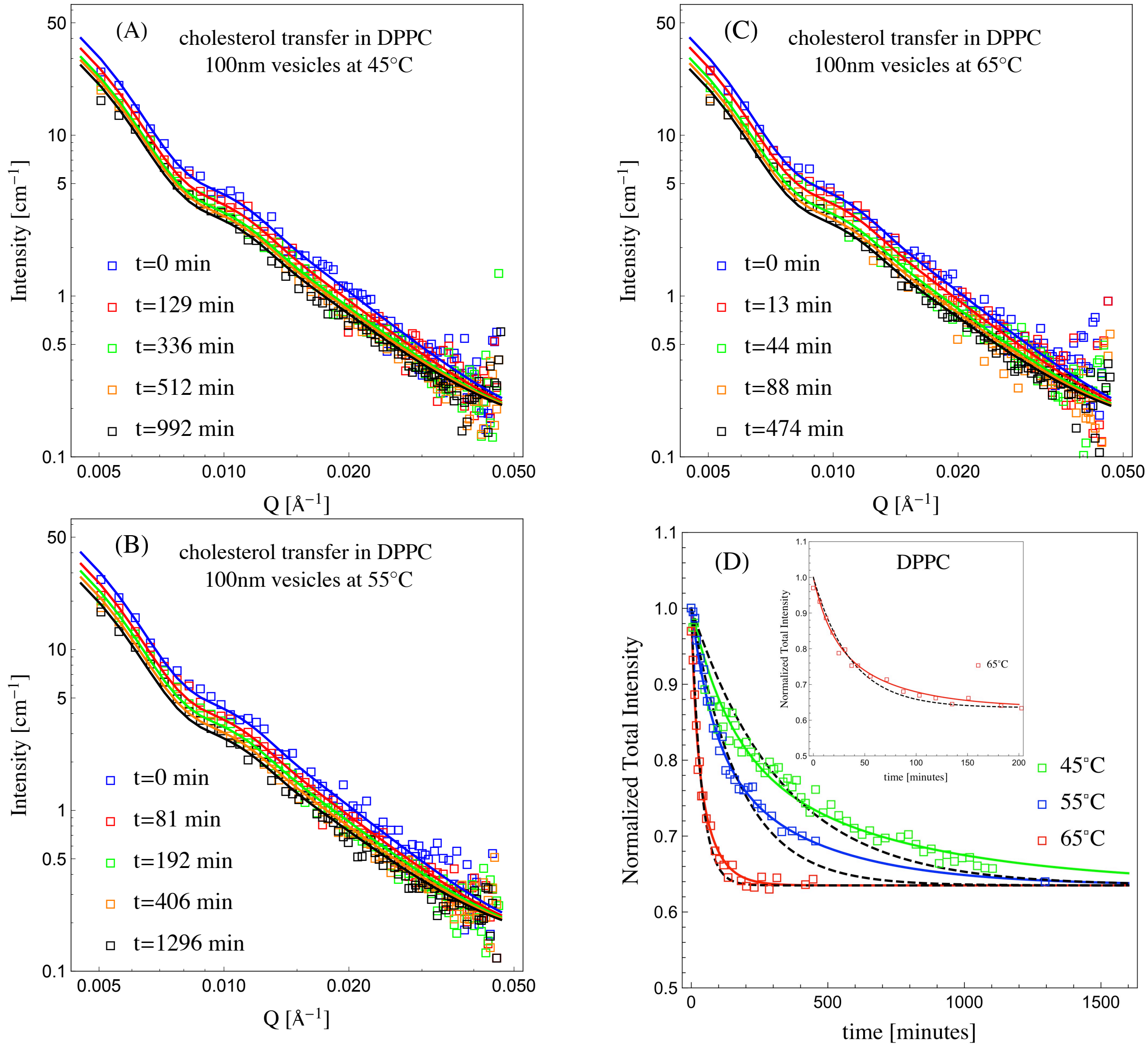

Figure S1. Scattered intensities as a function of time tracking the movement of cholesterol between donor and acceptor dDPPC vesicles at (A) 45°C, (B) 55°C and (C) 65°C. (D) Resulting normalized total intensities as a function of time for these three temperatures. Fits to the normalized total intensity were done with Equation 1 and from which flipping and exchange rates were extracted. Also shown in (D) are fits (black dashed lines) in which flipping is not rate limiting and only the exchange process is considered. The inset shows the 65°C data in a shorter time range to highlight the fits. Corresponding calculated scattering curves using Equation 2 are plotted in (A), (B) and (C). The form factor of the vesicles is obtained from the measurement of just the donor population.

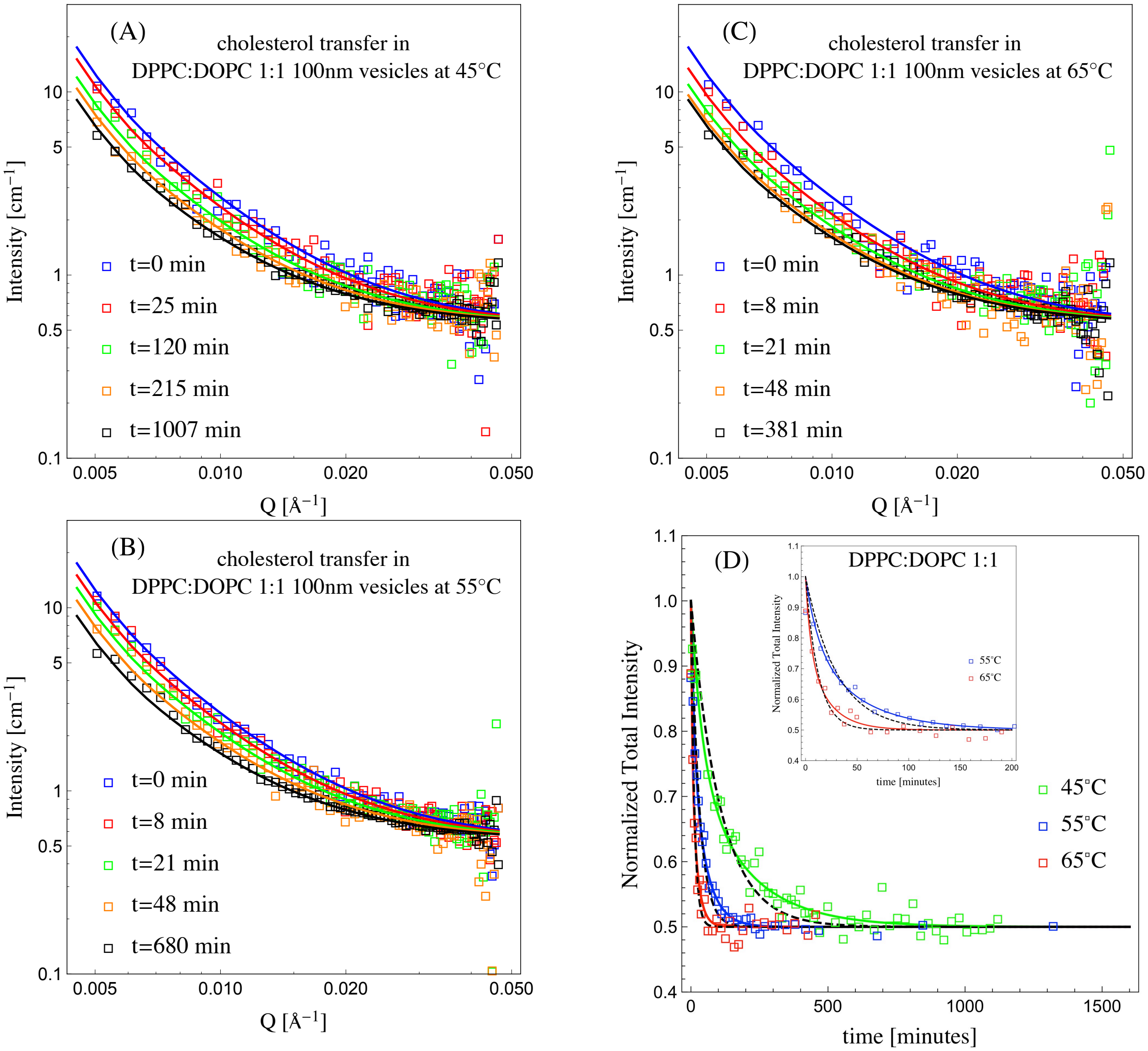

Figure S2. Scattered intensities as a function of time tracking the movement of cholesterol between donor and acceptor dDPPC:DOPC 1:1 vesicles at (A) 45°C, (B) 55°C and (C) 65°C. (D) Resulting normalized total intensities as a function of time for these three temperatures. Fits to the normalized total intensity were done with Equation 1 and from which flipping and exchange rates were extracted. Also shown in (D) are fits (black dashed lines) in which flipping is not rate limiting and only the exchange process is considered. The inset shows the 55°C and 65°C data in a shorter time range to highlight the fits. Corresponding calculated scattering curves using Equation 2 are plotted in (A), (B) and (C). The form

factor of the vesicles is obtained from the measurement of just the donor population.

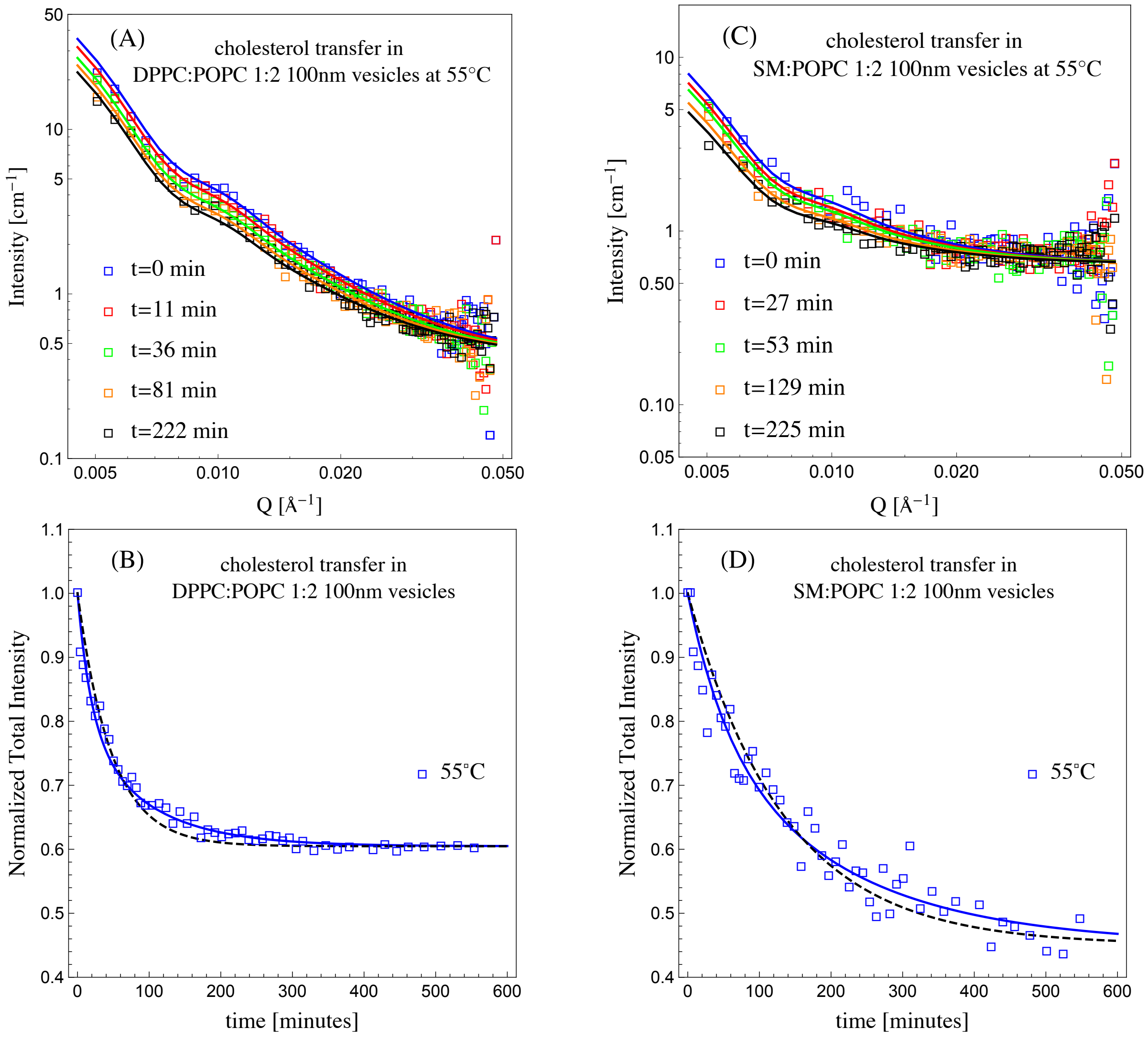

Figure S3. Scattered intensities as a function of time and corresponding normalized total intensity at 55°C tracking the movement of cholesterol between donor and acceptor vesicles with lipid compositions dDPPC:dPOPC 1:2 in (A) and (B) and PSM:dPOPC 1:2 in (C) and (D). As previously, fits to the normalized total intensity were done with Equation 1 and from which flipping and exchange rates were extracted. Also shown in (B) and (D) are fits (black dashed lines) in which flipping is not rate limiting

and only the exchange process is considered. Corresponding calculated scattering curves using Equation 2 are plotted in (A) and (C). The form factor of the vesicles is obtained from the measurement of just the donor population.

DPPC	$AIC_{flip\ and\ ex}$	AIC_{ex}	Likelihood that the only exchange model has equivalent information to the flip-flop and exchange model: $e^{(AIC_{flip\ and\ ex} - AIC_{ex})/2}$
45°C	-288.134	-238.617	$2x10^{-11}$
55°C	-174.994	-145.788	$4x10^{-7}$
65°C	-137.392	-127.109	$6x10^{-3}$

DPPC:POPC 1:1	$AIC_{flip\ and\ ex}$	AIC_{ex}	Likelihood that the only exchange model has equivalent information to the flip-flop and exchange model: $e^{(AIC_{flip\ and\ ex} - AIC_{ex})/2}$
45°C	-336.648	-259.207	$1x10^{-17}$
55°C	-190.22	-154.199	$2x10^{-8}$
65°C	-144.075	-138.129	$5x10^{-2}$

DPPC:DOPC 1:1	$AIC_{flip\ and\ ex}$	AIC_{ex}	Likelihood that the only exchange model has equivalent information to the flip-flop and exchange model: $e^{(AIC_{flip\ and\ ex} - AIC_{ex})/2}$
45°C	-247.395	-226.462	$3x10^{-5}$
55°C	-146.079	-138.005	$2x10^{-2}$
65°C	-111.607	-109.029	$3x10^{-1}$

55°C	$AIC_{flip\ and\ ex}$	AIC_{ex}	Likelihood that the only exchange model has equivalent information to the flip-flop and

			exchange model: $e^{(AIC_{\text{flip and ex}} - AIC_{\text{ex}})/2}$
DPPC:POPC 1:2	-273.025	-237.74	2x10^{-8}
SM:POPC 1:2	-196.859	-182.947	1x10^{-3}

Table S1. AIC values for each fit obtained through the built-in function in the software Mathematica™
